# Preparation and Characterization of Freely-Suspended Graphene Nanomechanical Membrane Devices with Quantum Dots for Point-of-Care Applications

**DOI:** 10.3390/mi11010104

**Published:** 2020-01-18

**Authors:** Gorkem Memisoglu, Burhan Gulbahar, Ruben Fernandez Bello

**Affiliations:** 1 Department of Communications Engineering, University of the Basque Country (UPV/EHU), Plaza Ingeniero Torres Quevedo 1, E-48013 Bilbao, Spain; ruben.fernandez@ehu.eus; 2 Department of Electrical and Electronics Engineering, Ozyegin University, 34794 Istanbul, Turkey; burhan.gulbahar@ozyegin.edu.tr

**Keywords:** graphene oxide, graphene, quantum dot, nanomechanical membrane, acoustic sensing, VFRET, point-of-care

## Abstract

We demonstrate freely suspended graphene-based nanomechanical membranes (NMMs) as acoustic sensors in the audible frequency range. Simple and low-cost procedures are used to fabricate NMMs with various thicknesses based on graphene layers grown by graphite exfoliation and solution processed graphene oxide. In addition, NMMs are grafted with quantum dots (QDs) for characterizing mass sensitive vibrational properties. Thickness, roughness, deformation, deflection and emissions of NMMs with attached QDs are experimented and analyzed by utilizing atomic force microscopy, Raman spectroscopy, laser induced deflection analyzer and spectrophotometers. Förster resonance energy transfer (FRET) is experimentally achieved between the QDs attached on NMMs and nearby glass surfaces for illustrating acousto-optic utilization in future experimental implementations combining vibrational properties of NMMs with optical emission properties of QDs. This property denoted as vibrating FRET (VFRET) is previously introduced in theoretical studies while important experimental steps are for the first time achieved in this study for future VFRET implementations. The proposed modeling and experimental methodology are promising for future novel applications such as NMM based biosensing, photonics and VFRET based point-of-care (PoC) devices.

## 1. Introduction

Nanomechanical membranes (NMMs) are important with their impressive precision and sensitivity for the applications in engineering and science, such as; mass, gas, stress, acoustic or viscosity detecting systems, impermeable barrier layer, water purification or memory [1,2,3,4,5,6,7,8,9,10,11]. In addition, NMMs have great potential to be used in novel nanoengineering systems such as; vibrating Förster resonance energy transfer (VFRET) based point-of-care (PoC) device systems or acousto-optic transducer systems [6,12,13,14,15].

NMM material, thickness, size or the load capacity are important for its mechanical performance [10]. Graphene is a good candidate for being a nanoscale membrane material by means of its fascinating mechanical properties such as; high strength, elasticity, low weight, low residual stress and large breaking strength [16,17,18,19,20]. There are various practical methods to prepare graphene based NMMs: mechanical exfoliation, chemical vapor deposition or solution processed liquid phase reduction of graphene oxide [21,22,23,24,25]. Among these methods, when the target size of graphene layer is not more than a few tens of micrometers, facile and low-cost mechanical exfoliation and solution processed graphene oxide can be used [22,24,25,26].

Mechanics of NMMs subjected to point or pressure loads are analyzed in the literature in terms of the relationship between load and deflection [27,28]. In addition, the effects of load and acoustic pressure on the mechanical behavior of graphene and graphene oxide NMM mechanic behaviors are reported where the deflection distances are defined for clamped thin and thick NMMs [6,8,9,10]. However, loaded-NMMs are promising to be perfect molecular rulers to measure distances in nanoscale FRET systems. In addition, NMMs are loaded with donor quantum dot (QD) molecules for realizing VFRET with the acceptor QD molecules coated as another layer under the external sound pressure as a nanoscale acoustic and mass sensor [6,12,13,14,15]. VFRET promises important future applications as wireless acousto-optical transduction-based nanoscale sensors in diverse areas of technology [6,12,13,14,15].

FRET for QD based solid systems is firstly reported in 1996 [29]. Furthermore, separated donor and acceptor bilayer structures are analyzed since 2001 for applications such as; graded energy structures [30,31,32], and energy transfer mechanisms including novel NMM device system with VFRET [6,12,13,14,15]. Morphologic, spectroscopic and optic properties of the donor, acceptor, (donor attached NMM) and (donor attached on NMM + acceptor coated glass) structures are important for characterization and optimization of the novel VFRET based devices, as discussed in our previous theoretical studies [6,12,13,14,15]. The notation (A + B) denotes the surface-to-surface mechanical connection between the layers A and B throughout the text. In this study, experimental characterization of such graphene based NMM structures and FRET experiments are achieved as fundamental steps for future implementations of VFRET.

The objective of this study is to present the freely suspended acoustically vibrating graphene-based pristine and QD loaded NMM preparations, their morphologic, spectroscopic and mechanical characterizations, and discussion on the possible energy transfer mechanism between the materials for utilization in PoC devices. NMM structures made of graphene are compared regarding their morphology, thickness, roughness, deformation, disorder-level and deflection including the load material effects. QD nanomaterials are grafted on NMMs as the load material while analyzing acoustically vibrated loaded-NMM deflections of NMMs with the loaded QD mass under varying pressure levels in the audible spectrum to form acousto-optic transducers. Various NMMs are manufactured and tested in terms of their vibrational and mechanical properties.

## 2. Materials and Methods

### 2.1. Materials

Graphite flakes (1 mm width) were purchased from HQ-graphene (Groningen, The Netherlands). Deionized water (DIW), acetone, and 2-propanole were supplied from Proquinorte S.A. (Bilbao, Spain). Graphene oxide hydrosol, and white/blue adhesive-tapes were from Graphenea (San Sebastian, Spain), 3M Inc. (Two Harbors, MN, USA) and Nitto Inc. (Osaka, Japan), respectively. CdTe (cadmium telluride) QD nanocrystals with different diameters were provided from PlasmaChem (Berlin, Germany). Donor CdTe and acceptor CdTe diameters were 2.2 nm and 3.1 nm, respectively. Microscope glass slides (1 mm thick) were purchased from Thermo Fisher Scientific (Braunschweig, Germany) and drilled for the width of 30 µm with femtosecond (fs) laser at SGIker (Bilbao, Spain) laser facility of the UPV/EHU.

### 2.2. Graphene Many Layer and Graphene Oxide Layer Sample Preparations

Graphene many layer (GML) samples were prepared by a mechanically exfoliation process of graphite similar to the one reported in [33]. Graphite flakes were mechanically exfoliated by adhesive tapes multiple times to prepare samples in varying thicknesses. For example, five times by low-adhesive blue tape and then by a chemically stable, strong-sticky, non-flexible white adhesive tape for nine, seven, five and three times exfoliations were applied for the samples a, b, c and d, respectively. More than nine times exfoliation decreased the GML size to less than 35 µm. Thus, maximum nine-times exfoliation was used for the experiments. Solution processed graphene oxide layer (GOL) samples were prepared with the stock hydrosol solution of the 10–15 µm size graphene monolayers (4 mg/mL). Sonication (Nahita Akralab, Alicante, Spain) was used for 10 min before the sample preparation, against the tendency to produce aggregates. Graphene oxide hydrosol blends (graphene oxide: DIW) were prepared with 1:0; 1:1; 1:2 and 1:3 by weight as samples e, f, g and h, respectively. Each sample with the same volume was drop casted onto clean and flat glass substrates and kept in an oven for 1 h at 100 °C for the preparation of GOL. To prevent the thermally unstable behavior of the graphene oxide, temperature was fixed at 100 °C while removing the DIW from the GOL [34]. More than 1:3 graphene oxide hydrosol blend ratio decreased the concentration to less than 1 mg/mL. Thus, the maximum 1:3 blend ratio was used for the experiments. It should be noted again that the samples a, b, c and d belong to the GML sample-set in the order of 9-, 7-, 5- and 3-times exfoliations, respectively, while samples e, f, g and h belonged to the GOL sample-set with graphene oxide: DIW with the ratios of 1:0; 1:1; 1:2 and 1:3 by weight, respectively.

### 2.3. Suspended NMM and Loaded-NMM Preparations

**Figure 1:**
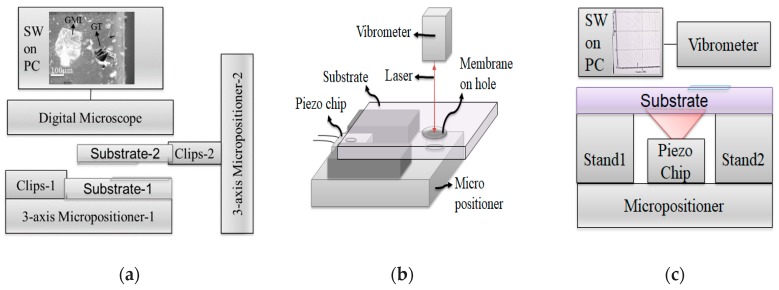
(**a**) Block diagram of the membrane transfer set-up with a microscope image of graphite (GT) and graphene many layer (GML) on glass; (**b**) deflection measurement system set-up and (**c**) deflection measurement system block diagram.

Prepared GML and GOL samples were transferred onto micron-size holes (30 µm width) in glass substrates, to be experimented as NMMs, with a practically implemented opto-mechanical transfer set-up whose block diagram is shown in Figure 1a, which was similar to the studies in [33,35]. In the set-up, a digital microscope (Dinolite, Leverstock, UK), micro-positioners and clips (Thorlabs, Munich, Germany) were used to adjust the membrane position on the hole and to fix the substrate samples during the transfer-process. In Figure 1a, graphite (GT) and GML morphologies were shown taken by a digital microscope connected to the membrane transfer set-up. Wet and dry stamp-transfer techniques were used for the GML and GOL, respectively [33,35]. Chemically soluble white adhesive-tape was used during the stamp-transfer process for the suspended GML preparation. The target substrate (attached onto the micropositioner-2) was wetted by chemical solvents (acetone: isopropanol; 1:2) for melting the adhesive of the tape and removing the layers from the tape surface onto the hole. In addition, suspended GOL preparation was performed by using of mechanical lithography and dry stamp-transfer method [33,35]. GOL and GML samples were utilized as NMMs after transferring onto the holes, in this study. Suspended NMMs were stretched in tension on holes without using any clamping by means of the interlayer adhesion energy (Van-der-Waals bonds) between the graphene and the glass substrate [23,36,37,38]. CdTe QDs with a 2.2 nm diameter were used as the load material for realizing the loaded-NMM, which is denoted as (donor CdTe attached on NMM) in this manuscript. Various concentrations of CdTe in methanol between 50 and 1.5 µM were prepared and the same volume (5 µL) of CdTe sample was drop-casted onto the NMMs to change the mass amount on the NMM and then to analyze the morphology, optical, spectroscopic and mechanical properties of the loaded-NMM.

### 2.4. NMM Characterizations, Performance Analysis, Loaded-NMM Deflection and Spectroscopic Experiments

In order to characterize the surface morphology, thickness and roughness of the samples, atomic force microscope (AFM, Bruker, Billerica, MA, USA) was used at the peak-force mode (down to 10 pN), for a 1 Hz scan rate. Deformation and disorder levels of the samples are analyzed with a confocal Raman spectroscopy system (Renishaw, Gloucestershire, UK) with a 532 nm laser, 1% power and 10 s integration time. NMM and loaded-NMM (donor CdTe attached on NMM) deflection measurements were performed with an acoustic actuator chip - laser Doppler vibrometer system whose set-up is shown in Figure 1b. In the set-up; there existed an acoustic actuator chip (Thorlabs) placed at 1 cm fixed distance under the NMM sample for providing sound pressure in the audio frequency range; a micro-positioner (Thorlabs) to define the measurement point as the middle of the NMM and a picometer (pm) distance sensitive laser Doppler vibrometer (Polytec, Coventry, UK) to measure the deflection distance of the membrane by sending and collecting 630 nm laser light and processing the signal between 20 Hz and 20 kHz. An external voltage-amplifier with a function generator (Keysight, Santa Rosa, CA, USA) was used to drive the acoustic actuator chip at various sound pressures and frequencies. A digital multimeter (Keysight) and a digital sound level meter (Bafx, Scottsdale, AZ, USA) were used to adjust the voltage value and the sound pressure. The general block diagram of the deflection measurement is shown in Figure 1c. Deflections were recorded from the center of each NMM. Spectrometric characterizations of the materials and the layers, i.e., donor and acceptor CdTe, acceptor CdTe, GML, GOL, (donor CdTe attached on NMM) and (donor CdTe attached on NMM + acceptor CdTe coated glass) were performed by using a multimode UV-VIS absorption and emission plate reader, a UV-VIS absorption spectrophotometer and a spectrometer (Biotek Instruments, Winooski, VT, USA, Carry Agilent Technologies, Santa Clara, CA, USA, and Ocean Optics, Largo, FL, USA, respectively).

## 3. Results and Discussion

**Figure 2:**
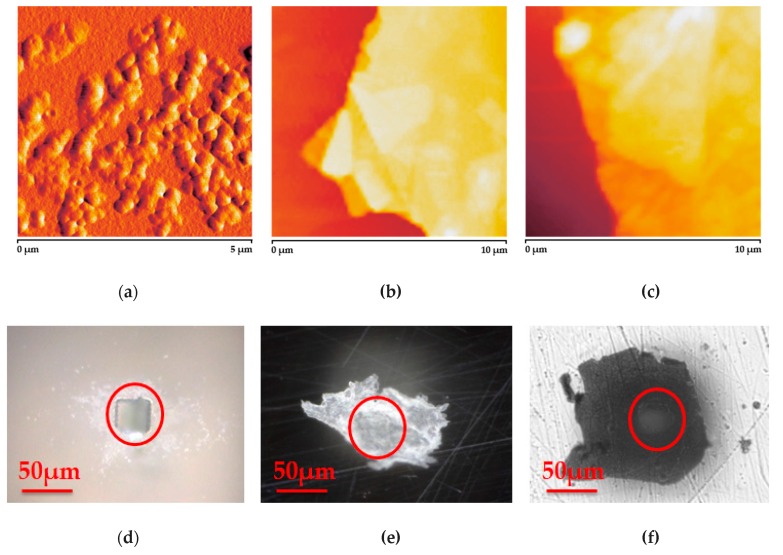
Atomic force microscope (AFM) images of: (**a**) the load quantum dot (QD) material, (**b**) GML, (**c**) graphene oxide layer (GOL) and digital microscopy images of: (**d**) drilled substrate, (**e**) suspended GML and (**f**) suspended GOL, respectively. Red-circles in figures (**d**–**f)** correspond to the drilled areas where the membranes are suspended.

Thickness, roughness, deformation, surface morphology and deflection distances of NMM (with and without the load material), and the mass, roughness, surface morphology and emission intensity of the attached QD material were important parameters for the performance optimization in various applications such as photovoltaic devices, sensors, and NMM based novel acoustic sensors [1,2,3,4,5,6,7,8,9,10,11,12,13,32] as proposed in this article. Microscopy images of the prepared NMMs are shown in Figure 2, where Figure 2a–c and Figure 2d–f belonged to the atomic force and optical microscopy images, respectively. Figure 2a shows the surface morphology of drop casted and annealed 1.5 µM CdTe QD with the thickness of 7.4 nm. Discrete dispersion of the 0.2–0.5 µm (width) clusters of the 2.2 nm diameter CdTe was homogeneous with the surface roughness of 5.8 nm. In Figure 2b, an exfoliated GML image is shown with the thickness of 80 nm on glass substrate. In addition, graphene flakes with the size less than 10 nm are visible in the 0.5 µm thick GOL film sample on glass in Figure 2c. Figure 2d shows the 30 µm drilled substrate before covered by an NMM. 

Suspended 80 nm thick GML and 0.5 µm thick GOL images are shown in Figure 2e,f, respectively. Red-circles in Figure 2d–f correspond to the drilled areas where the membranes are suspended.

Thickness and roughness values of the exfoliation time dependent GML, and hydrosol blend ratio dependent GOL samples are given in Table 1. Thickness values are in the ranges of 80–325 nm, and 118–1493 nm for GML and GOL samples, respectively. GML samples have roughness values between 109 and 150 nm for the first seven-times exfoliations (samples d, c and b). However, after seven-times exfoliation times (sample a), roughness decreases three-times in consequence of the decrease in the layer numbers (around a few ten layers). For the GOL samples, the one with the weight ratio of 1:1 by weight has at least 10 times less roughness than the sample layer without DIW. 

The resulting measurements of the thickness of hundreds of nanometers are highly suitable to attach a large amount of QD molecules on the membranes to increase the light emission intensity from the PoC sensor verifying the simulation studies in [6,13,14]. The practical manufacturing combined with the capability of massive QD loading provide a future promising PoC sensor material technology based on VFRET mechanism.

**Figure 3:**
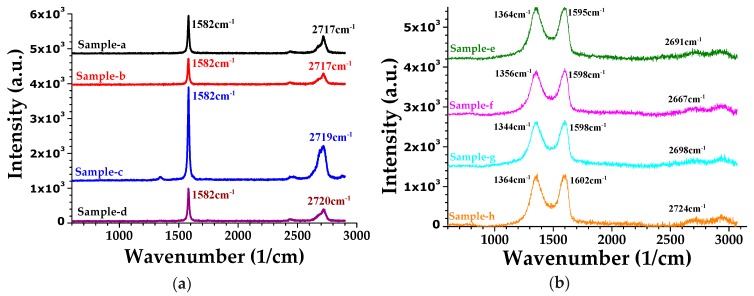
Raman peaks of: (**a**) GML (samples a–d), and (**b**) GOL (samples e–h); λ_exc_ = 532 nm.

Raman spectroscopy is used to analyze the deformation and disorder levels of the NMM. Wavenumber shifts, intensity and the locations of G-peak, D-band, D’-band and 2D-band of samples can give information on the deformation and defect levels [39,40,41]. Characteristic graphene peaks of the produced NMM samples are shown in Figure 3. Graphene G-peaks are located at 1582 cm^−1^ for the GML samples with no wavenumber shifts. However, G-peaks of the GOL samples shifted to the longer-wavenumbers from 1595 to 1602 cm^−1^ with increasing DIW amount in the graphene oxide: DIW blends, i.e., from sample e to sample h. In addition, G-peak and D-band intensity ratio (*I_D_/I_G_*) of the GML and GOL samples were in the ranges of 1.19–0.04 and 1.04–0.97, respectively. Increase in the *I_D_/I_G_* ratio was related to less deformation and decreased defect level in graphene-based samples [39]. According to the *I_D_/I_G_* ratios, GOL samples presented slightly larger *I_D_/I_G_* ratio than the GML samples (except the thinnest ones). The reason of the decreased defect and deformation level of the GOL samples is attributed to the sp^2^ C atoms, which surround the defects [39]. Furthermore, characterization by using the intensity ratio between the D-band and the G-peak is not suitable for the quality comparison of the GOL samples due to the superposition of G-peak and D’-band, and the reasons that the measured G-peak is the sum of the both G-peak and D’-band [39]. 

2D-band shift analysis is another helpful way for the sample quality comparison, where the information of 2D-band shifting to the larger wavenumbers is responsible for the disordered (more amorphous) nature and defective behavior [40]. 

2D-bands are located around 2717–2720 cm^−1^ for the GML and 2667–2724 cm^−1^ for GOL samples. In the GOL samples, the minimum 2D-band shift is at the sample f, which suggests to the lower disorder nature and defective behavior with the lowest shift in 2D-band [40]. Moreover, the higher-wavenumber shift in the 2D-band is attributed to the increasing layer number of a sample [41], which is compatible with the GML samples from sample a to sample d. The resulting measurements of the Raman spectrum verified that the graphene-based nature was preserved for practical implementations of NMMs for the PoC sensor design. This shows that the material and experimental manufacturing methods were suitable for future prototypes while allowing tuning the sample quality by optimizing the defect level based on analyzing the Raman peak intensity ratios (*I_D_/I_G_*) or shifts ($S_{2D}$), as shown in Table 1.

**Figure 4:**
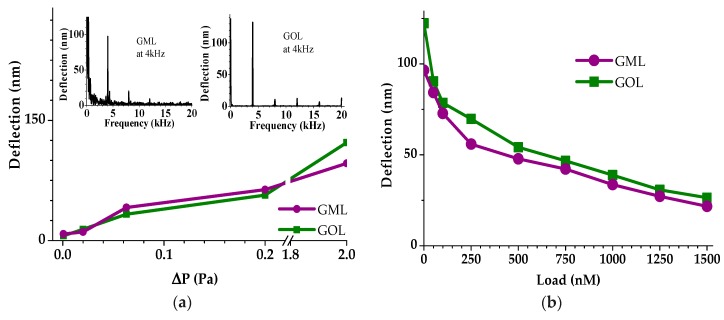
(**a**) Deflection as a function of the applied sound pressure (ΔP). The inset figures show the mechanic resonance spectrum for GML and GOL membranes at 2 Pa and (**b**) deflection of NMMs with increasing load at 2 Pa.

Deflection experiments are important to understand the pristine NMM and loaded-NMM device responses against applied sound pressures in audible frequency range [10,42,43,44,45,46]. Figure 4a shows the applied sound pressure (ΔP) dependent NMM deflections. Deflection distance increased with increasing ΔP values for the both GML and GOL. The NMM deflections increase with increasing ΔP, between 54.1 and 97.5 nm for the GML, and 70–132 nm for the GOL samples, respectively, with increasing ΔP while the pressure was between 0.02 and 2 Pa (Figure 4a, and Table 1).

The inset figures in Figure 4a show the frequency dependent NMM deflection characteristics at 2 Pa sound pressure. Strong-peaks at 4 kHz were in correlation with the acoustic source frequency, while the periodic small-peaks at multiples of 4 kHz such as 8 kHz and 12 kHz were the harmonic signals. These harmonic peaks were attributed to the presence of the degenerate modes whose degeneracy has been lifted by asymmetries in either the surface contamination or stress profile of the NMM [42]. The deflection amounts of tens of nanometer are enough to induce VFRET emissions requiring more than ten nanometers to switch FRET On and Off. On the other hand, observations of both the deflection for proposed sound pressure levels much less than 1 Pa and operation at the audible frequency range of several kHz provide a practical implementation environment for future acousto-optic PoC sensor systems. As future work, we will perform measurements for diverse types of membranes classifying their responses in terms of pressure sensitivity, acoustic frequency and bandwidth.

Deflection is proportional to the exfoliation number, and inversely proportional to the thickness for the GML samples, while the deflections of GOL samples do not follow the thickness dependency due to the other material effects inside its structure such as epoxy, carboxyl or carbonyl [10]. Thus, it is crucial to optimize the graphene oxide hydrosol blend ratio for preparing a better GOL as NMM.

Prepared GOL sample deflections are decreasing from 132 to 86.9 nm by increasing dilution ratios. The reason of the decrease in the NMM deflection with increasing dilution is the decreasing concentration of functional groups such as; carboxyl and epoxy, which are responsible for the stretching effects and the elasticity in the blend [10]. In addition to this, non-diluted GOL (sample e) has the lowest deflection distance in the GOL sample-set, due to its high roughness and thickness.

Loaded-NMM (donor CdTe attached on NMM) analysis shows that the deflection slightly decreases with increasing membrane concentration from 50 to 1.5 µM, as shown in Figure 4b. The reason for the decrease in the deflection is the increasing total mass and thickness of the system [9,44]. The measurement results show that the dilution of GOL samples was a practical method to tune roughness, thickness and the desired deflection for future PoC sensor implementations.

It was observed that increasing membrane load and decreasing applied sound pressure, both resulted in a decrease in the deflection distances, which means that the produced devices were capable of scaling both the mass of the air and the attached molecules on the NMM surface.

**Figure 5:**
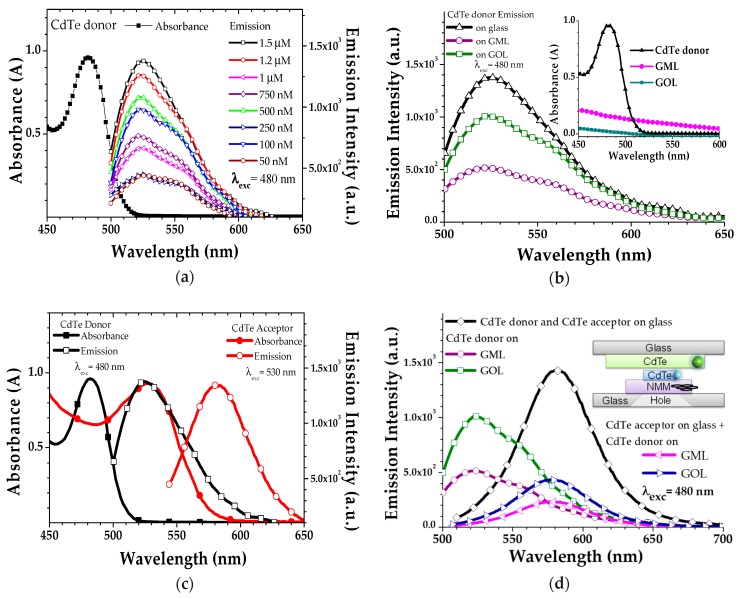
(**a**) Absorption and emission spectra of CdTe donor with the concentration dependent emission intensity characteristics on glass slides; (**b**) emission characteristics of (donor CdTe attached on GOL) and (donor CdTe attached on GML) under 480 nm excitation light. Inset figure corresponds to the self-absorption characteristics of the CdTe donor, GML and GOL; (**c**) absorption and emission spectra with the spectral overlap (shaded area) of CdTe donor and CdTe acceptor materials and (**d**) emission characteristics of the connected (donor CdTe attached on NMM + acceptor CdTe coated glass) devices with the comparison of (donor CdTe attached on NMM). Inset image shows the general (donor CdTe attached on NMM + acceptor CdTe coated glass) device structure where the CdTe donor attached on NMM is connected directly to CdTe acceptor coated glass. Emission intensities in a, b and d are collected for the λ_exc_ = 480 nm.

Donor CdTe had the absorption and emission maximums at 480 nm and 530 nm, respectively, as shown in Figure 5a. Absorption peak and concentration dependent emission characteristics of drop-casted donor CdTe on glass samples are shown in Figure 5a where the maximum emission was with the 1.5 µM concentration sample, under 480 nm donor excitation light. 

However, at larger concentrations more than 1.5 µM, emission quenches dramatically (not shown) due to the aggregation of the material with the high concentration [45,46]. Donor CdTe with 1.5 µM concentration was selected for performing the (donor CdTe attached on NMM) characterizations. In Figure 5b, emission intensity characteristic was compared between the pristine donor CdTe and the donor CdTe loaded-NMMs to understand the interaction between donor CdTe and NMM under donor CdTe excitation light (480 nm). Emission characteristic in Figure 5b shows that CdTe emission was quenched when it was grafted onto NMM due to the energy transfer from fluorophores of CdTe to the graphene based NMM [19,47,48,49,50,51,52]. However, when we compared the emission quenching intensities, emission quenching rate of CdTe attached on GML sample emission quenching rate was higher than the CdTe attached on GOL sample.

Same type (CdTe) QDs with different diameters (2.2 nm donor, and 3.1 nm acceptor) were chosen to experiment FRET process from small-size dot (donor) to the big-size dot (acceptor). 

Bandgap of the small-size donor dot (2.2 nm CdTe) was larger than the big-size acceptor dot (3.1 nm CdTe), due to the reciprocal proportion of the bandgap energy with the nanocrystal size [52,53,54].

Figure 5c shows the absorption and emission spectra with the spectral overlap region between emission of donor CdTe and absorption of acceptor CdTe where the FRET occurs when the dipole-dipole coupling is provided and molecular distance is less than 10 nm between donor and acceptor molecules under donor excitation light [13,19,49,50,51,52,53,54].

Emission characteristics are shown in Figure 5d for the (donor CdTe attached on GML + acceptor CdTe coated glass), and (donor CdTe attached on GOL + acceptor CdTe coated glass) under donor excitation light. General (donor CdTe attached on NMM + acceptor CdTe coated glass) device structure is shown in the inset of Figure 5d, where the continuous donor excitation light was introduced from the bottom-side of the device, while the acceptor emission was collected from the top-side. 

Under the donor excitation light, the (donor CdTe attached on NMM + acceptor CdTe coated glass) device emitted at 585 nm wavelength, which belongs to the acceptor CdTe emission maximum. This means that an energy transfer occurred between the two connected systems from donor CdTe to the acceptor CdTe. However, emission intensities of the (donor CdTe attached on NMM + acceptor CdTe coated glass) devices were lower than the (donor CdTe attached on NMM) emission intensities. This device will be optimized under various sound pressure by adjusting the distance between donor and acceptor CdTe molecules by developing a new set up where the (donor CdTe attached on NMM) layer was fixed and the (acceptor CdTe coated glass) layer was approached through the fixed part, in nanometer steps as future work.

**Figure 6:**
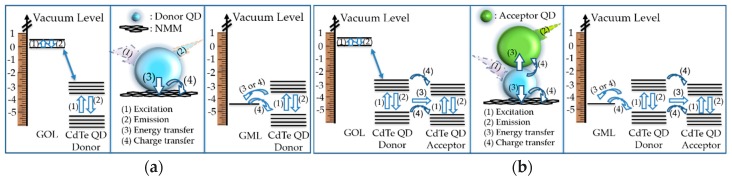
Energy level of the materials with the proposed mechanism for the relationship between: (**a**) donor CdTe and GOL, and donor CdTe and GML; (**b**) (donor CdTe attached on GOL) and (donor CdTe coated glass), and (donor CdTe attached on GOL) and (donor CdTe coated glass).

In Figure 6a,b, proposed mechanisms and schematic illustrations with energy levels, energy and charge transfers are shown for defining the relationship between the materials [19,29,49,50,51,52,53,54]. Step (1) of the mechanism is the excitation of the materials under the donor CdTe excitation light. Step (2) is the emission of CdTe, and step (3) is the nonradiative energy transfer. The final step (4) is the charge transfer. There was 1 eV distance between the energy level of GML and donor CdTe CB (conduction band); while a large energy level distance (3.1 eV) was present between donor CdTe CB and the HOMO (highest occupied molecular orbital) level of the GOL [19,29,49,50,51]. The more energy level distance between CdTe donor and GOL compared with CdTe donor and GML means that there is stronger possibility of the more nonradiative energy transfer and charge transfer between CdTe donor and GML, whilst higher donor CdTe emission quenching was obtained with GML compared with GOL. In addition, due to the harmony in the energy levels (Figure 6b), and spectral overlap region (Figure 5c), there were strong energy and charge transfer possibilities when there was (acceptor CdTe coated glass), which had sensitivity to the emission of donor CdTe and placed at a fixed distance less than 10 nm from (donor CdTe attached on NMM) under donor CdTe excitation light. The perfect spectrum overlapping and the tuning of emission intensities verified the proposed materials and methods for practical implementations of PoC sensors targeting specific emission capability.

## 4. Future Application of VFRET for PoC Sensor System

**Figure 7:**
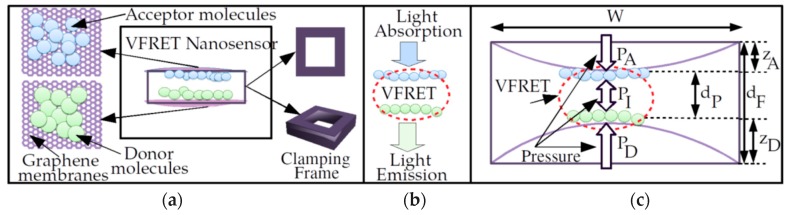
(**a**) Nanoscale sensor design based on VFRET with two graphene membranes clamped on a frame where donors and acceptors are inside a cage of impermeable graphene layers, (**b**) absorption of light by donors at the frequency of donor excitation and emission from acceptor after VFRET between donors and acceptors and (**c**) pressure dependent variation in inter-membrane distance [6,14,15].

The proposed PoC nanoscale sensor (NS) or a special kind of molecular machine design is shown in Figure 7a. It is composed of two membranes, donor and acceptor molecules and a frame holding the membranes tightly. Graphene membranes are impermeable and do not allow other molecules to pass and interact with donors and acceptors chemically or mechanically [5,55] by forming a cage. The simple mechanical system allows the transduction of the acoustic vibrations or instantaneous strain due to external forces to periodic or strain based constant amplitude optical signals from the acceptor molecules, i.e., VFRET, as shown in Figure 7b,c, with ambient light energy at the excitation frequency of the donor shining on the device.

Optical modulation speed can reach tens of GHz with fast radiative lifetimes of FRET and fast resonance frequencies obtained with smaller sensors without any complicated optical modulation circuitry in [13,14]. It is an advanced and stand-alone version of the fundamental architecture proposed in [6,12,13,14,15].

The simple and purely mechanical NS with the weight on the orders of femtogram to picogram (at least $10^{3}$ times smaller than the weight of average human cell) promises novel applications such as optical nanoscale tagging and signal generation, microfluidic particle tracking and real-time and nanoscale resolution flow monitoring, e.g., air and liquid. 

The important features of the NS in comparison with competitive designs for monitoring are listed as follows:

- It is fully mechanical, stand-alone and with planar architecture based on strong and low weight 2D graphene based material, donor–acceptor couples such as QDs with atomic scale dimensions and a frame, which could be designed by carbon material [14,15]. The simplicity and strong mechanical structure provide long lifetime and durability of the sensor properties.
- The NS is mobile, flexible and impermeable to external molecules keeping the donors and the acceptors intact. It allows attachment without invasive destruction of the material properties due to mobility and low weight such as on biological substances, drugs or molecular flows in liquid or air.
- VFRET NS provides a tagging mechanism by improving fluorescence imaging-based systems with optical signaling capability especially for in-body and microfluidic biomedical imaging by exploiting time-varying optical emission sequences [14,15]. Graphene is nearly transparent allowing the two-way optical transmission in the challenging medium such as inside human cells.
- It has the capability of high-speed monitoring for real-time pressure measurement of air and liquid flows medium converting the instantaneous pressure differences to optical emissions.
- The NS do not require complicated electronics hardware or software for energy storage and signal modulation while harvesting the external pressure and ambient light at the excitation frequency of donor. Next, significant properties of the NS are utilized in the PoC and the environmental monitoring applications compared with the state-of-the-art.

In this study, experimental characterization of NMM structures and FRET experiments were performed as building blocks of PoC sensor system. On the other hand, implementation of the complete architecture is a future work requiring precise verification of VFRET mechanisms between the membrane attached QD molecules. Furthermore, clamping frame manufacturing and attachment of the membranes with high precision are important challenges. We provided a highly feasible and practical PoC sensor design by also providing the experimental implementations of the fundamental building blocks, i.e., practical NMM structures. The implementation of PoC sensor was closer in the near future based on the proposed design and experimental studies in this article.

## 5. Conclusions

NMM systems based on graphene suspended layers were prepared, experimented and analyzed to study how graphene-based layers could serve as audible frequency acoustic sensing and mass sensitive nanomechanical devices. According to the experimental studies, freely suspended clamp-free micron-size quantum dot loaded graphene based NMMs act as kHz-range acoustic sensing layers with deflection distances on the order of nanometers. In addition, quantum dot donor and acceptors were used and FRET was achieved in the nanomechanical device system where the acceptor quantum dot light emission was obtained from the (donor CdTe attached on NMM + acceptor CdTe coated glass) device structure under the donor excitation light. This work is an important contribution to be used in the diverse applications both in air or in microfluidic studies including photoacoustic-based imaging in spectroscopy, the VFRET based acousto-optic transducer devices and light sensor systems as the future works.

## Figures and Tables

**Table 1:** Nanomechanical membrane (NMM) characteristics ^1^.

Number Sample Detail		*t* (nm)	*R* (nm)	*I_D_/I_G_*	*S*_2*D*_ (cm^−1^)	*D* (nm)
a	9 times graphite exfoliation	80	38.6	1.19	2717	97.5
b	7 times graphite exfoliation	265	150	0.63	2717	93.7
c	5 times graphite exfoliation	268	142	0.45	2719	81.7
d	3 times graphite exfoliation	325	109	0.04	2720	54.1
e	graphene oxide:DIW; 1:0	1493	179	1.00	2691	70
f	graphene oxide:DIW; 1:1	556	17.3	0.99	2667	132
g	graphene oxide:DIW; 1:2	208	16.5	0.98	2698	103
h	graphene oxide:DIW; 1:3	118	10.9	1.03	2724	86.9

^1^t is the thickness, R is the roughness, $I_{D}/I_{G}$ is the Raman peak intensity ratio of the D-band and G-peak, $S_{2D}$ is the Raman 2D-peak position and D is the experimentally obtained deflection distance (deflections were recorded at 2 Pa).
